# The PhenX Toolkit: Long COVID Collection of Standard Protocols

**DOI:** 10.1002/cpz1.70317

**Published:** 2026-04-22

**Authors:** Michelle C. Krzyzanowski, Helen Pan, Iris Glaze, Stephen Hwang, David Williams, Michelle Engle, Justin Waterfield, Cataia Ives, Sara Armson, Wayne Huggins, Carol M. Hamilton

**Affiliations:** ^1^ RTI International Durham North Carolina; ^2^ Current affiliation: Office of the National Coordinator for Health Information Technology Washington District of Columbia

**Keywords:** COVID‐19, Data harmonization, Long COVID, PhenX Toolkit, SARS‐CoV‐2, Variable Compare Tool

## Abstract

Because of the urgent need to better understand the severe acute respiratory syndrome coronavirus 2 (SARS‐CoV‐2) transmission mechanism and clinical outcomes resulting from infection, health organizations and research institutes developed data collection instruments to assess coronavirus disease 2019 (COVID‐19) symptoms, treatments, and associated impacts on behaviors and risks, treatment and outcomes, information available, psychosocial and mental health, and socioeconomic effects across demographic groups. The PhenX team developed the COVID‐19 Protocol Library to share data collection instruments, methods, and protocols in use by investigators and to reduce proliferation of similar protocols. The Office of Behavioral and Social Sciences Research and the National Human Genome Research Institute sponsored the effort, in collaboration with the NIH Disaster Research Response Program. As the COVID‐19 library was being established, crowdsourcing was used to identify key topics related to the pandemic, which formed the basis of the COVID‐19 Research Collection, and was released in the PhenX Toolkit. Long COVID continues to have an impact on human health. Long COVID is a chronic condition that occurs after SARS‐CoV‐2 infection and is present for at least 3 months, encompassing a variety of symptoms or conditions that may improve, worsen, or be ongoing. Not surprisingly, submissions to the COVID‐19 library began to address Long COVID symptoms. After discussions with its Steering Committee, the PhenX team established a collection of Long COVID measurement protocols. Also, there was significant interest in assessing the amount of overlap among all instruments submitted to the COVID‐19 library. The intent was to develop a tool that could identify potential for cross‐study analyses. The COVID‐19 Variable Compare Tool (VCT) integrates all COVID‐19 Research Collection protocols and a prioritized subset of instruments from the COVID‐19 library. The Long COVID Specialty Collection and the VCT support investigators’ needs to combine and analyze data related to COVID‐19 prospectively and retrospectively by identifying compatibility with other studies. © 2026 RTI International. Current Protocols published by Wiley Periodicals LLC.

**Basic Protocol**: Exploring Long COVID through PhenX Toolkit Specialty Collections and the Variable Compare Tool

## INTRODUCTION

**Figure 1:**
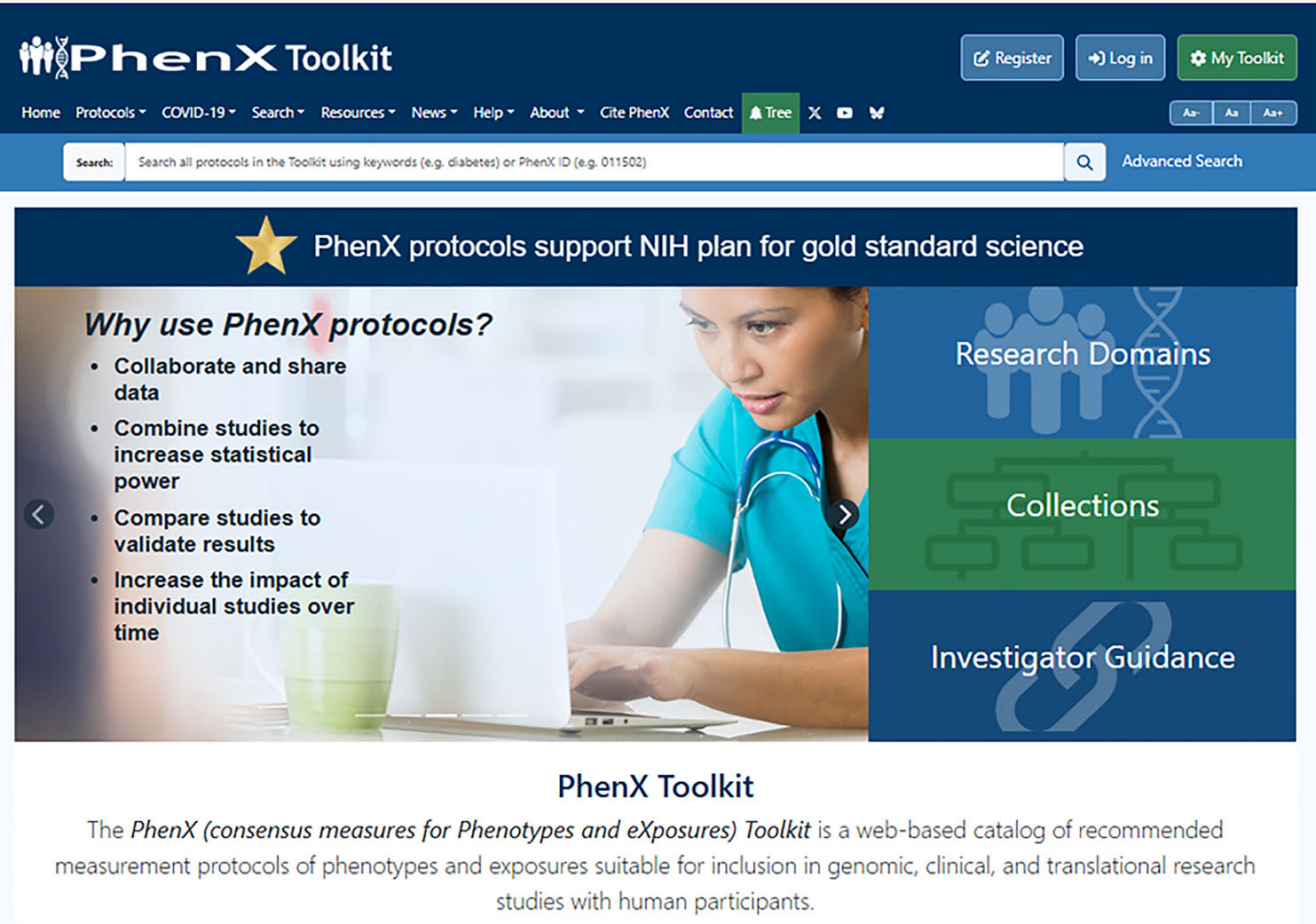
The PhenX Toolkit home page and top navigation menu.

In March 2020, the World Health Organization released an official declaration that COVID‐19 had become a pandemic (Cucinotta & Vanelli, 2020) As part of the “NIH‐Wide Strategic Plan for COVID‐19 Research”, the PhenX (consensus measures for Phenotypes and eXposures) team collaborated with the NIH Office of Behavioral and Social Sciences Research, the National Human Genome Research Institute, and the NIH Disaster Research Response (DR2) Program to create a protocol library of COVID‐19‐related data collection instruments (see Internet Resources). New SARS‐CoV‐2 variants demonstrated increased transmissibility and resistance to vaccines (Raman et al., 2021). The instruments added to the COVID‐19 Protocol Library (see Table 1), acquired from NIH from 2020 through 2022, included hospital intake forms and medical history, as well as questionnaires to assess the impact of COVID‐19 on daily life. The far‐reaching impacts included education, finances, mental health, and food security (Krzyzanowski et al., 2021), with different emphasis, depending on the study. Using protocols from this library, feedback from the community through crowdsourcing, and overarching guidance from the PhenX Steering Committee (SC), the COVID‐19 Research Collection was released in the PhenX Toolkit to allow researchers to combine and compare studies with increasing ease (Krzyzanowski et al., 2021). The COVID‐19 Research Collection (see Internet Resources) can be reached by clicking “COVID‐19 Research Collection” from the dropdown in the top navigation menu (Fig. 1). As the COVID‐19 rates decreased and vaccinations became more widespread, new knowledge of Long COVID symptoms (Davis et al., 2023), i.e., chronic ailments that continued after having the condition, became investigators’ focus. Investigators began submitting Long COVID protocols to the COVID‐19 Protocol Library, and the research focus shifted to addressing Long COVID symptoms and quality of life. As a result, we worked with the PhenX SC through discussion, review, and approval to add a Long COVID Specialty Collection and expand the larger COVID‐19 Research Collection, continuing the COVID‐19 project work. The new Long COVID Specialty Collection (see Internet Resources) can be accessed through the COVID‐19 Research Collection landing page.

**Table 1:** PhenX COVID‐19 Project Concepts and Terminology

Concept	Definition
COVID‐19 protocol library	Provides COVID‐19‐related measurement protocols (case report forms, data collection forms, instruments, surveys, questionnaires) that are currently in use
Full instrument	The full‐length protocol or questionnaire submitted by the original authors
Module	A section or portion of a full instrument; these subject‐related sections are suggested by the authors and the NIH; PhenX creates modules from the full instruments and assigns subtopics
Instrument source	The authors of the full instrument
Protocol	A standard data collection procedure, or measurement protocol, recommended by a PhenX Working Group
Specialty Collection	Complementary to a core collection; provides more in‐depth assessments of a specific topic or field of research

The COVID‐19 Research Collection provides useful protocols to collect key information related to the COVID‐19 pandemic, but an investigator may have additional study needs. The COVID‐19 library has 382 protocols, covering data collection instruments from USA‐ and international‐based studies. When adding these instruments to the library, the PhenX team noted that similar topic areas were often covered across different instruments, often with the purpose of collecting the same data. If further research sought to discover new insights into the effects of the COVID‐19 pandemic, combining these data collected across instruments would require instruction on how to harmonize the data (the practice of combining different datasets to maximize their comparability or compatibility) and ultimately facilitate cross‐study analysis and inform study design. Our next step was the creation of a COVID‐19 Variable Compare Tool (VCT) (see Internet Resources). From the COVID‐19 library, the PhenX team selected 26 instruments as part of this pilot study to examine the level of overlap among these data collection tools. These instruments came from large, well‐recognized studies [e.g., the Environmental influences on Child Health Outcomes (ECHO) Program] or from national or international health organizations [e.g., World Health Organization (WHO), U.S. Centers for Disease Control and Prevention (CDC)]. The PhenX team explored the degree of overlap among the tools and to analyze the level of similarity, and the resulting data were integrated into the VCT web application. These additions expanded the utility of COVID‐19 offerings from PhenX, contributing to NIH's goal to “facilitate the use of COVID‐19 data to the greatest extent possible” (Office of Data Science Strategy, 2020).

The COVID‐19 VCT provides three functions for investigators to explore the subject areas covered in these instruments in further depth than browsing the library alone, with the goals of facilitating cross‐study analysis of variables in COVID‐19‐related instruments and informing study design: (1) Keyword Search to find variables or questions containing those keywords, (2) Side‐by‐Side Comparison to directly compare two instruments and their amount of cross‐over, and (3) Compare Instruments and PhenX Protocols to visually gain insight into the similarity within a group of protocols. Investigators can reach the COVID‐19 VCT by clicking the COVID‐19 Variable Compare Tool link from the “COVID‐19” dropdown in the top navigation menu (Fig. 1). The VCT was designed to enable researchers to compare COVID‐19 data collection instruments at the protocol or variable level and identify opportunities for cross‐study analysis.

## STRATEGIC PLANNING

### Long COVID Specialty Collection

Of the full instruments in the COVID‐19 Protocol Library, 64% were added in 2020, leading to the initial creation of the COVID‐19 Research Collection, which was released in October 2020. Another 21% were added in 2021 and another 15% through July 2022. Long COVID–related instruments were added to the library from October 2021 to July 2022. In late 2021, the PhenX team proposed expansion of the COVID‐19 Research Collection to include a Long COVID collection of protocols. In January 2022, the PhenX SC approved inclusion of protocols that addressed Long COVID and COVID‐19 vaccination. As a result, the PhenX team identified modules from instruments being used by Researching COVID to Enhance Recovery (RECOVER), the University College London, the Fred Hutchinson Cancer Center, and the Kaiser Family Foundation. We created REDCap (Research Electronic Data Capture) surveys for the SC to review and provide feedback on the modules, which covered Long COVID symptoms, impact on daily life, and vaccination.

The first round of feedback focused on identifying the best choices across these topics. Ultimately, SC members and the PhenX team agreed that in many cases, having brief and more detailed protocols would be beneficial to investigators. This ultimately led to the creation of “short‐form” (brief) and “long‐form” (detailed) protocols. The second round of feedback focused on the name of the collection, the higher‐level categories, and the content associated with each category. Ultimately, 27 new protocols were added to the PhenX Toolkit, which included 25 protocols in the new Long COVID Specialty Collection and two vaccination protocols in the existing COVID‐19 History, Treatment and Outcomes Specialty Collection.

### Variable Compare Tool

The COVID‐19 Protocol Library contains 185 instruments, divided into 867 modules by topic (e.g., economic effects, mental health) and associated with up to three descriptive subtopics. For this pilot study, we prioritized a subset of the instruments based on NIH recommendations. For the development of the VCT, prioritized instruments (1) were created by major studies and sources, including ECHO, the Adolescent Brain Cognitive Development (ABCD) Study, CDC, and WHO; and (2) covered a wide range of topics, thus increasing the chance of finding overlapping variables across instruments.

### Data Dictionaries and Mapping Process

To establish the variable data mappings for the VCT, we extracted data collection instrument variables from source files and converted them into a standard data dictionary format. The finalized versions of the data dictionaries were fed into an R Shiny tool to make comparing variables easier. We conducted a variable mapping process (in which we compared variables between protocols) which enables investigators to identify linked variables across studies that were not retrievable using Keyword Search, thus enhancing the findability of linked datasets. Following previous PhenX mapping efforts (Pan et al., 2022), we defined three categories in which variables could be similar to one another, seen in Table 2. “Identical” variables are immediately ready for direct sharing or harmonization among datasets. “Comparable” mappings require a simple logical or mathematical transformation or categorical approximation or grouping before data can be combined. Finally, a “related” mapping level is assigned to variables that are conceptually related, but the context of the data collection is different. For example, related variables may refer to different recall periods, may collect the same information on different subjects (e.g., subject may be the patient vs patient's spouse), or may be useful for only a subset of responses. We further refined mapping rules through first finding variables with similar keywords (e.g., “symptoms,” “treatment”) and working through what qualified as identical, comparable, or related. Once these rules and examples were established, we completed mapping for all selected instruments from the COVID‐19 Protocol Library. Mapping went through a round of review to ensure consistency and quality. Any discrepancy was resolved in a group discussion.

**Table 2:** Mapping Types*^a^*

Mapping type	Description
Identical	Variables that are immediately ready for direct harmonization between datasets, without any transformation needed to combine the data
Comparable	Variables that are conceptually similar and that contain data that can be directly harmonized or compared after a simple logical or mathematical transformation
Related	Variables that have comparable concepts, but the conditions, such as the period of time over which the data are collected, of that concept are different

^*a*^ Similarity levels or “mapping types” between variables. “Mapping type” indicates the similarity level, whereas “description” provides base criteria meeting that similarity level.

**Figure 2:**
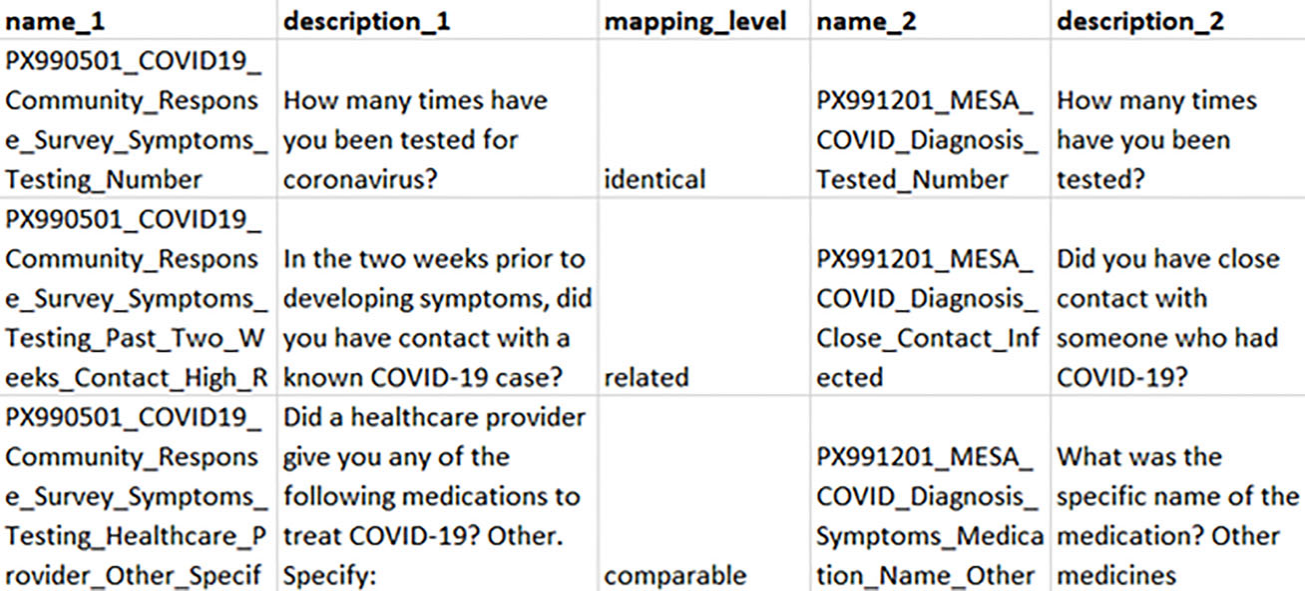
Examples of mappings at the identical, comparable, and related levels.

Figure 2 provides examples of each mapping level. The identical pair shows two variables with almost the exact same wording that capture the same concept: the number of COVID‐19 tests a respondent has had. The comparable pair shows how the same concept (medications to treat COVID‐19) is being captured in both questions but with different wording. The related pair shows how each question captures a similar, but not the same, concept. The core question asks if a respondent has had close contact with someone with COVID‐19; however, one of the questions includes a time qualifier of being within the past 2 weeks, whereas the other variable has no additional qualifiers.

### Long COVID Specialty Collection Results

The PhenX team created a Long COVID Specialty Collection, which was added as part of the larger COVID‐19 Research Collection (see Internet Resources). These cutting‐edge data collection protocols were added with a “rapid release” status, indicating our effort to make them quickly available to the research community. Primary considerations for selection of the recommended COVID‐19 protocols included being from a reputable source, broadly applicable to research and clinical assessment, and based on input from SC recommendations. Other considerations included availability in other languages, applicability to people of various life stages, and being available free of charge to the public.

The creation of the Long COVID Specialty Collection and addition of the COVID‐19 vaccination protocols fully integrate the rapid‐release COVID‐19 protocols into the PhenX Toolkit (see Table 3). That is, the Toolkit briefly describes each measurement protocol, the purpose and rationale for its inclusion, standard protocols, references, and requirements (e.g., training, personnel, equipment, applicable licensing fees) for data or specimen collection. Toolkit users can search or browse for these COVID‐19‐related protocols and save selected protocols in My Toolkit. Users can also download a custom Data Collection Worksheet (DCW) that details their selections and allows them to easily “cut and paste” protocols to incorporate them into their data collection study design documentation. Finally, users can also download a REDCap instance for data collection.

**Table 3:** New Long COVID Specialty Collection and New Vaccination Protocols

Specialty Collection	PhenX Protocols
Long COVID	Long COVID ‐ Comorbidities and COVID‐19 Impact Long COVID ‐ Current Quality of Life ‐ Child Long COVID ‐ Daily Living Long COVID ‐ Multisystem Inflammatory Syndrome ‐ Child Long COVID ‐ Physical Functioning Long COVID ‐ Symptoms Due to COVID‐19 ‐ Allergies Long COVID ‐ Symptoms Due to COVID‐19 ‐ Cardiovascular Symptom Course Long COVID ‐ Symptoms Due to COVID‐19 ‐ Gastrointestinal (Long Form) Long COVID ‐ Symptoms Due to COVID‐19 ‐ Gastrointestinal (Short Form) Long COVID ‐ Symptoms Due to COVID‐19 ‐ Genitourinary Long COVID ‐ Symptoms Due to COVID‐19 ‐ Memory Long COVID ‐ Symptoms Due to COVID‐19 ‐ Muscle and Joint Long COVID ‐ Symptoms Due to COVID‐19 ‐ Neurology Long COVID ‐ Symptoms Due to COVID‐19 ‐ Ocular (Long Form) Long COVID ‐ Symptoms Due to COVID‐19 ‐ Ocular (Short Form) Long COVID ‐ Symptoms Due to COVID‐19 ‐ Pediatric Long COVID ‐ Symptoms Due to COVID‐19 ‐ Psychiatric (Long Form) Long COVID ‐ Symptoms Due to COVID‐19 ‐ Psychiatric (Short Form) Long COVID ‐ Symptoms Due to COVID‐19 ‐ Psychological Risk Factors Long COVID ‐ Symptoms Due to COVID‐19 ‐ Respiratory Long COVID ‐ Symptoms Due to COVID‐19 ‐ Screener Long COVID ‐ Symptoms Due to COVID‐19 ‐ Skin and Hair Long COVID ‐ Symptoms Due to COVID‐19 ‐ Speech, Language, and Hearing Long COVID ‐ Symptoms Due to COVID‐19 ‐ Temperature Regulation and Cardiovascular Long COVID ‐ Symptoms Due to COVID‐19 ‐ Tooth Pain
History, treatment, and outcomes	COVID‐19 Vaccination ‐ Adult COVID‐19 Vaccination ‐ Child

### Mapping Results

The COVID‐19 VCT version 1.0 contained 19 COVID‐19 Protocol Library instruments and 20 COVID‐19 Research Collection protocols. Version 1.1 added another 7 COVID‐19 Protocol Library instruments focused on treatments and post‐COVID‐19.

**Figure 3:**
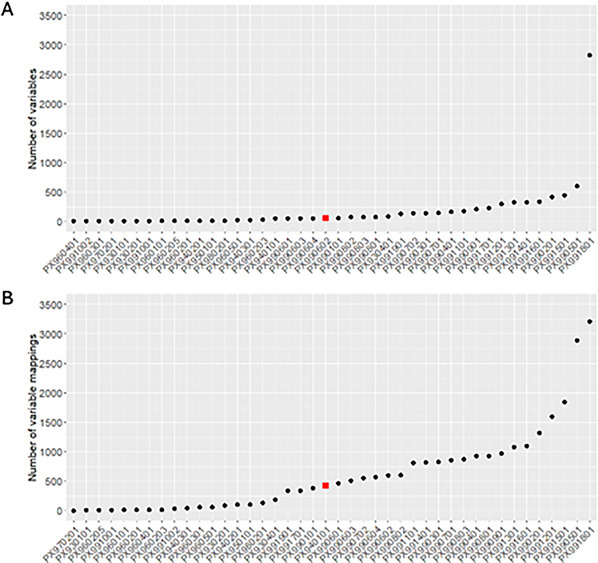
Number of variables per instrument or protocol. A total of 16 COVID‐19 Protocol Library instruments and 26 PhenX Toolkit COVID‐19 Research Collection protocols were used. (A) The number of variables per instrument ranged from 5 to 2822 variables (black dots), with a median of 62.5 variables per instrument (red square). Instrument PX990602 contains the median number of variables (*n* = 61) and is designated with a red square. (B) The number of mapped variables ranged from 2 to 3611 mappings (black dots), with a median of 466 mapped variables (red square) per instrument. Instrument PX940101 contains the median number of variables (*n* = 427) and is designated with a red square.

**Figure 4:**
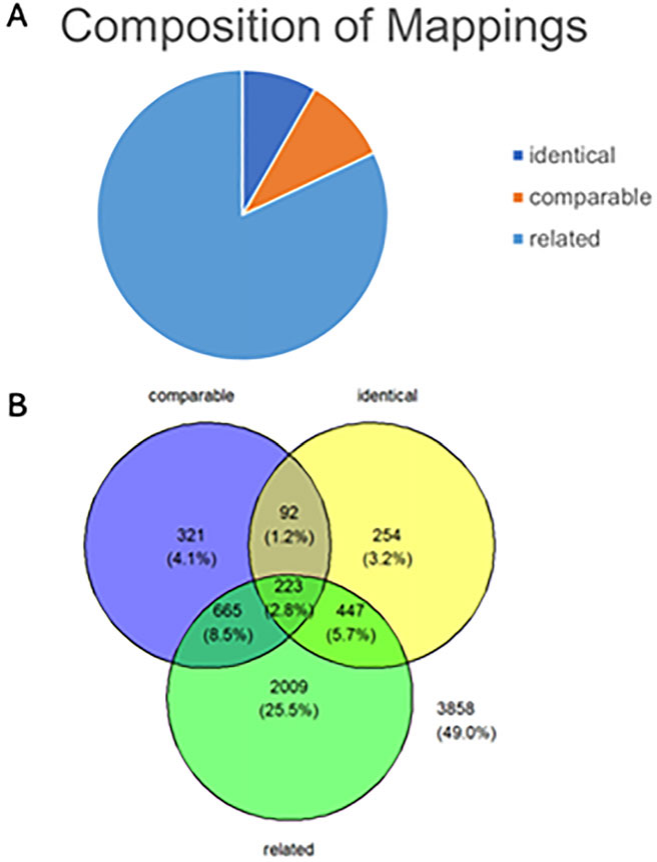
Mapping results from pilot set. (**A**) 1016 total mappings were at the identical level, 1301 were comparable, and 3344 were mapped as related variables. The total number of variables mapped was 7869. (**B**) A Venn diagram shows all variables from the instruments in the VCT and the mappings associated with them. There are 7869 variables in total. The 3 bubbles indicate variables that have comparable, identical, or related mappings associated with them (4011 variables, or 51.0% of all variables). Variables associated with more than one mapping level are indicated in the intersections of the bubbles. Variables that have no mappings associated with them (3858, or 49.0% of all variables) are indicated in the space outside the 3 bubbles.

The number of variables per instrument ranged from 5 to 2822, with a median of 62.5 variables per instrument, which influenced the number of similarities a given instrument could have with other instruments. The curation team mapped variables from one instrument to any conceptually related variables on other COVID‐19 instruments as outlined in “Data Dictionaries and Mapping Process”. After removing any variables mapped to variables in the same instrument, a total of 7869 variables were mapped to another variable through the mapping effort. Of these, 1016 (12.9%) total mappings were identical, 1301 (16.5%) were comparable, and 3344 (42.5%) were related variables. A total of 2208 variables (28%) were not mapped to any other variable and represent unique information captured (Figs. 3 and 4).

Of the 42 instruments, 29 consisted of variables that map at the identical level to another instrument and can therefore be used for directly comparing or combining data. 36 instruments contained variables that map at the comparable level, and 42 instruments contained variables mapped as related. 28 instruments contained variables mapped at all three levels (identical, comparable, and related). When looking at the number of mappings per instrument, there is again a wide range of variability. The number of mapped variables ranged from 2 to 3611 mappings, with a median of 466 mapped variables per English‐language instrument.

### Variables With the Most Mappings

The instrument that contains the most frequent mappings to variables in other instruments (3611 mappings) is the Yale COVID‐19 Recovery Study: Screening Questionnaire. Upon investigation, however, this instrument does not have the highest level of overlap with other instruments, and variables in this instrument map to variables on only 12 other instruments in the set. Interestingly, the Yale COVID‐19 Recovery Study: Screening Questionnaire was designed to focus primarily on symptoms related to COVID‐19 and post‐COVID‐19. Because of the variety of symptoms reviewed and the number of variables, there was a large number of mappings, but not as much overlap with other instruments due to the instruments’ narrow focus. This shows a limitation of this process due to the limitation of the source data.

The instrument that contains the second‐most mappings to variables in other instruments (2890 mappings) is Johns Hopkins University's (JHU's) COVID‐19 Community Response Survey. Upon investigation, this instrument had the highest level of overlap with other instruments, and variables in this instrument map to variables in a total of 26 other instruments within the set. Interestingly, the COVID‐19 Community Response Survey was designed to collate questions from several widely used survey sources and includes modules or subsets of questions from at least three other COVID‐19 Library instruments, including the American Life Panel Survey on Impacts of COVID‐19, the Understanding America Study (UAS), and the Collaborating Consortium of Cohorts Producing NIDA Opportunities (C3PNO) survey. The high level of overlap and interconnectivity among variables from the JHU COVID‐19 Community Response Survey and the other protocols in the PhenX COVID‐19 Research Collection therefore represents a logical confirmation of the curation process when the source data are topically diverse.

### Variable Compare Tool Results

The COVID‐19 VCT has three functions: (1) Keyword Search, (2) Side‐by‐Side Comparison, and (3) Compare Instruments and PhenX Protocols. The goal of these functions is for investigators to delve deeper into COVID‐19 instruments through the type of data being collected, topic areas covered, and level of similarity between instruments.

**Figure 5:**
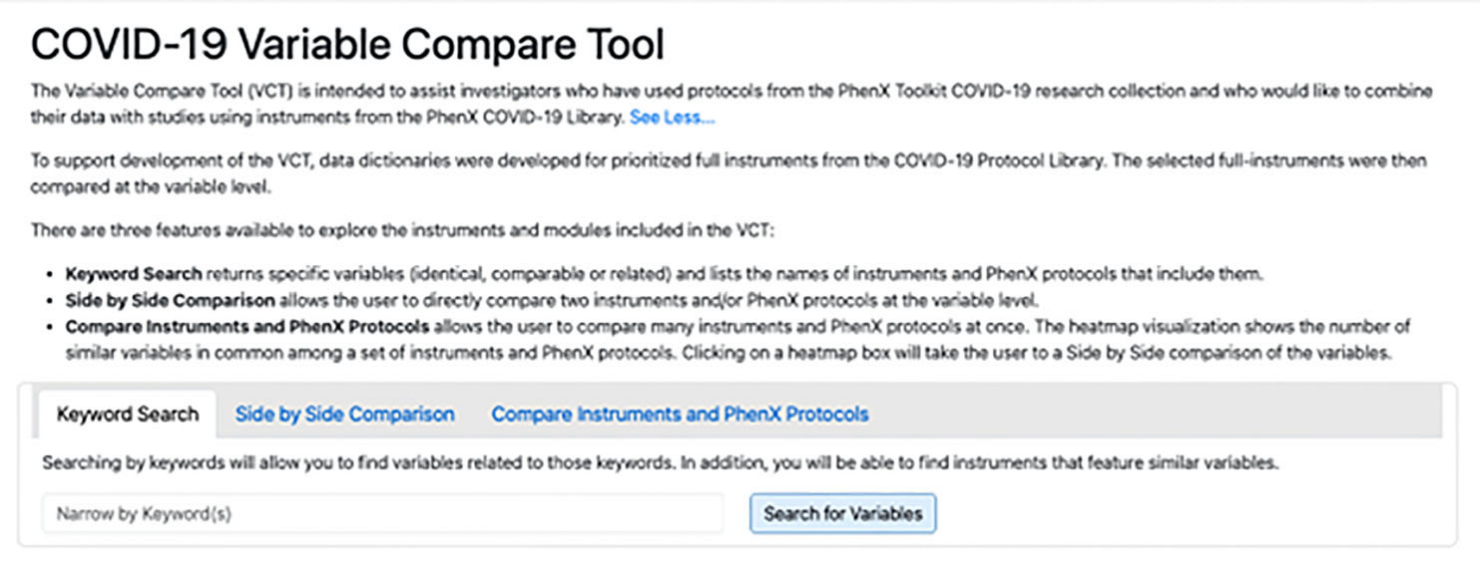
COVID‐19 Variable Compare Tool. The tool has three functions: Keyword Search, Side‐by‐Side Comparison, and Compare Instruments and PhenX Protocols. An investigator can access these functions by clicking on the tabs shown in this screenshot.

The Keyword Search (Fig. 5) takes the user‐input words and compares them with the text contained in the variables, i.e., description_1 and description_2, (see Fig. 2) for all instruments. If there is a partial or complete string match in the entire variable (“Description” column, Table 4), that variable is included in the returned array of positive matches. All matched variables are returned in a tabular format, with information on the variable code, variable description, and the instruments in which this variable, or a similar variable, can be found.

**Table 4:** Data Dictionary

Component	Description
VARNAME	Name for the variable
VARDESC	Variable description, question, or variable text
DOCFILE	Name of the data collection instrument file
TYPE	Data type of the variable
UNITS	Units of measurement of the variable
MIN	Minimum numerical constraint
MX	Maximum numerical constraint
COMMENT1	Any branching logic conditions that would prompt this variable to be used
VARIABLE_SOURCE	Source of the variable (i.e., PhenX)
SOURCE_VARIABLE_ID	Unique variable identification (ID) to ensure there are no duplicates
VALUES	List of encoded or enumerated values

**Figure 6:**
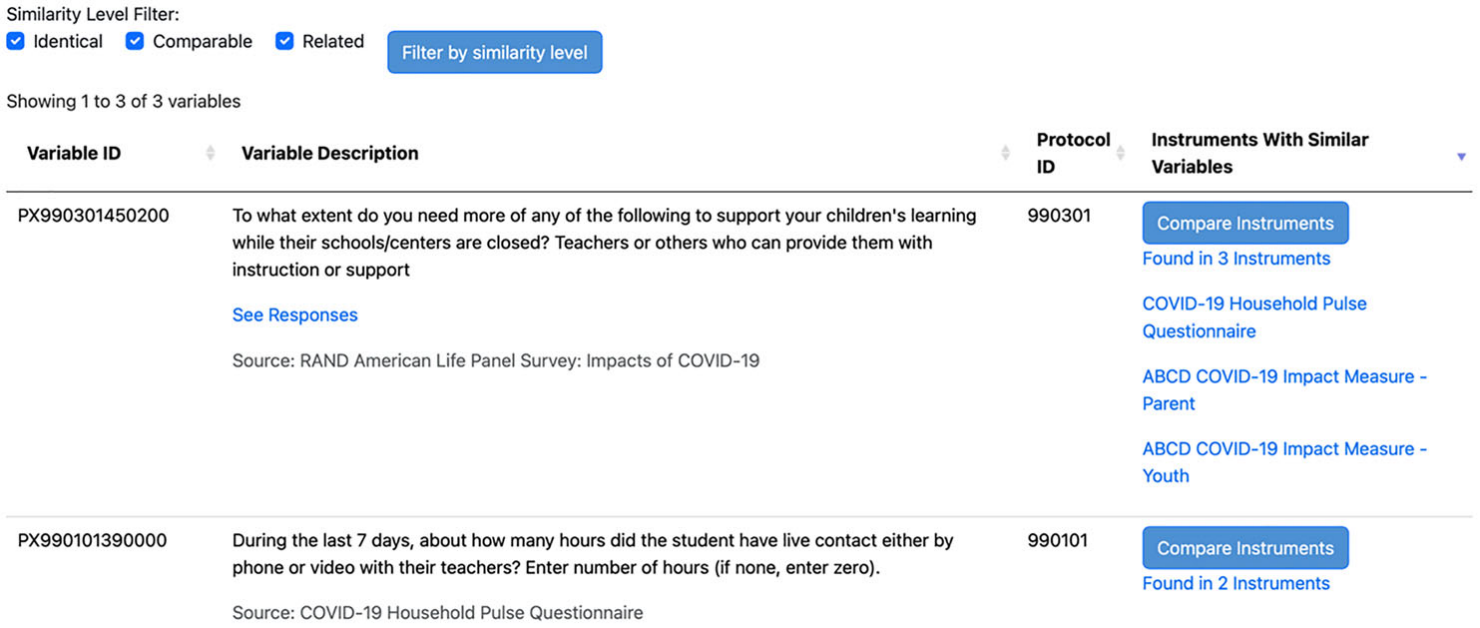
Example results from a keyword search.

The number of instruments with similar variables listed for each variable is determined by the mappings compiled and stored in the database from the aforementioned data dictionaries and mapping process based on the initial query. For each variable returned, data collection instruments listed have one or more variables that map as identical, comparable, or related to the matched variable listed. Results are returned in order of the number of instruments in which a similar variable is found. For example, a variable (i.e., the question) that matches the search term queried and has mappings to three instruments will be listed higher in the results than a variable that has mappings to two instruments (Fig. 6).

**Figure 7:**
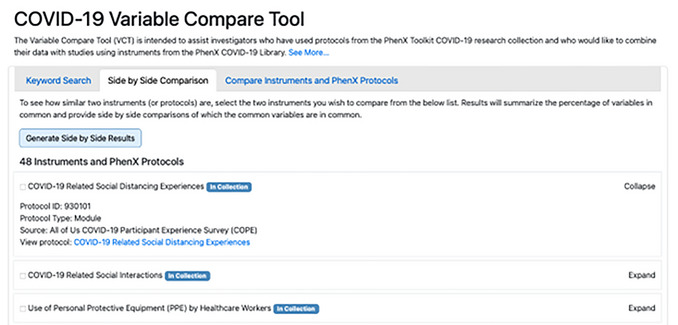
VCT Side‐by‐Side Comparison form. An investigator can select two instruments or COVID‐19 Research Collection protocols to generate results on how similar the selected instruments are and perform one‐to‐one matching between variables. Additional information about the instrument or protocol is provided as an expandable/collapsible view.

**Figure 8:**
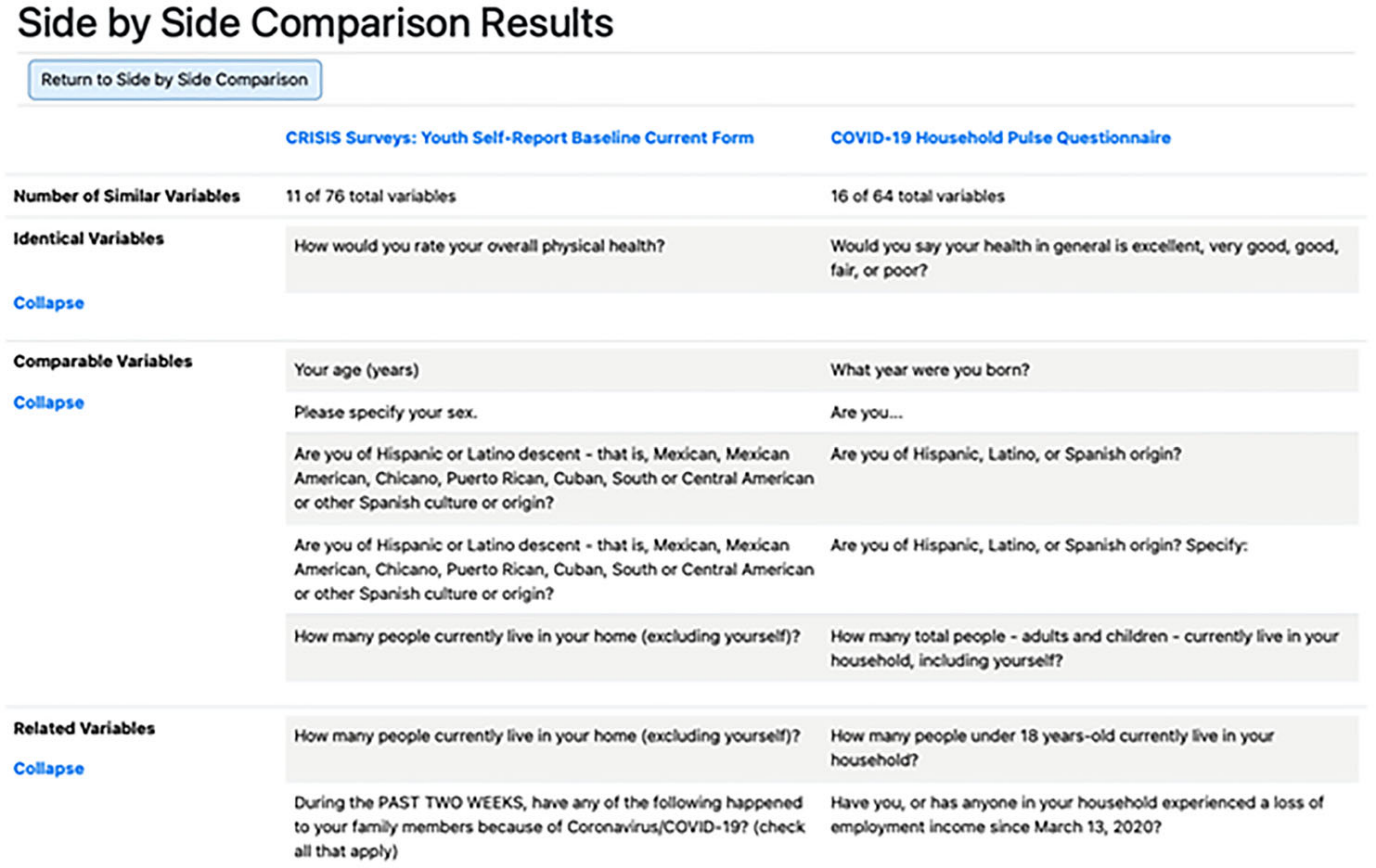
VCT Side‐by‐Side Comparison results. This illustrates a comparison between the CRISIS Surveys: Youth Self‐Report Baseline Current Form and the COVID‐19 Household Pulse Questionnaire. Similarities are broken down at the identical, comparable, and related levels.

The Side‐By‐Side Comparison allows an investigator to pick two instruments (labeled “In Library”) or PhenX COVID‐19 Research Collection protocols (labeled “In Collection”) and run a comparison of the two (Fig. 7). The results provide the similarities between the two selected instruments or protocols, with the number of variables that have at least one similarity and the breakdown of which variables are identical, comparable, or related (Fig. 8).

The results are organized into a three‐column table (Fig. 8). The second and third columns have data belonging to the data collection instrument or PhenX COVID‐19 Research Collection protocol, as indicated in the column header. The first column has additional descriptors for the data in the below rows. The data are organized to first show the number of variables mapped out of the total number of variables in each instrument or protocol. Then identical, comparable, and related variables are listed, side by side, to clearly indicate which variables map.

**Figure 9:**
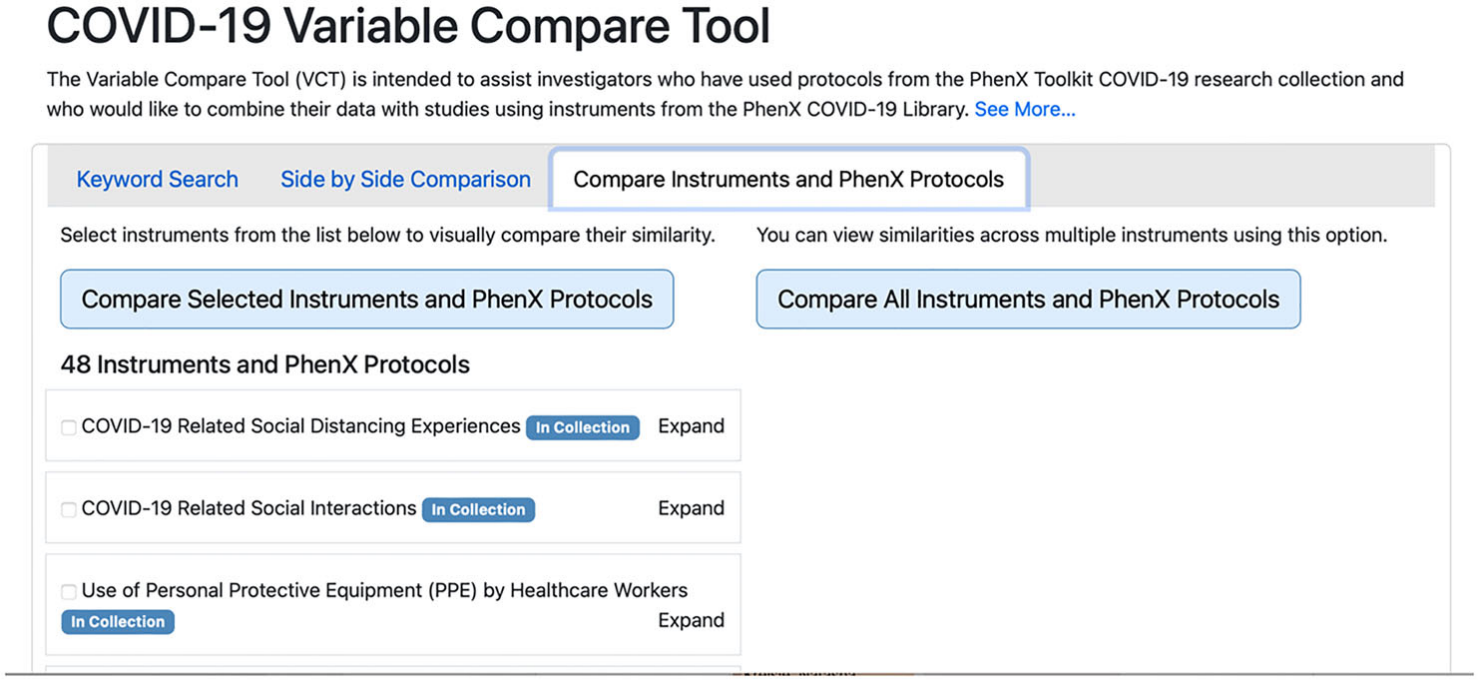
Compare Instruments and PhenX Protocols landing page.

The Compare Instruments and PhenX Protocols function has two options for the investigator. The “Compare All Instruments and PhenX Protocols” option provides a heatmap of the comparisons across all listed instruments and COVID‐19 Research Collection protocols. The “Compare Selected Instruments and PhenX Protocols” option generates a heatmap of investigator‐selected instruments or protocols to compare (Fig. 9).

**Figure 10:**
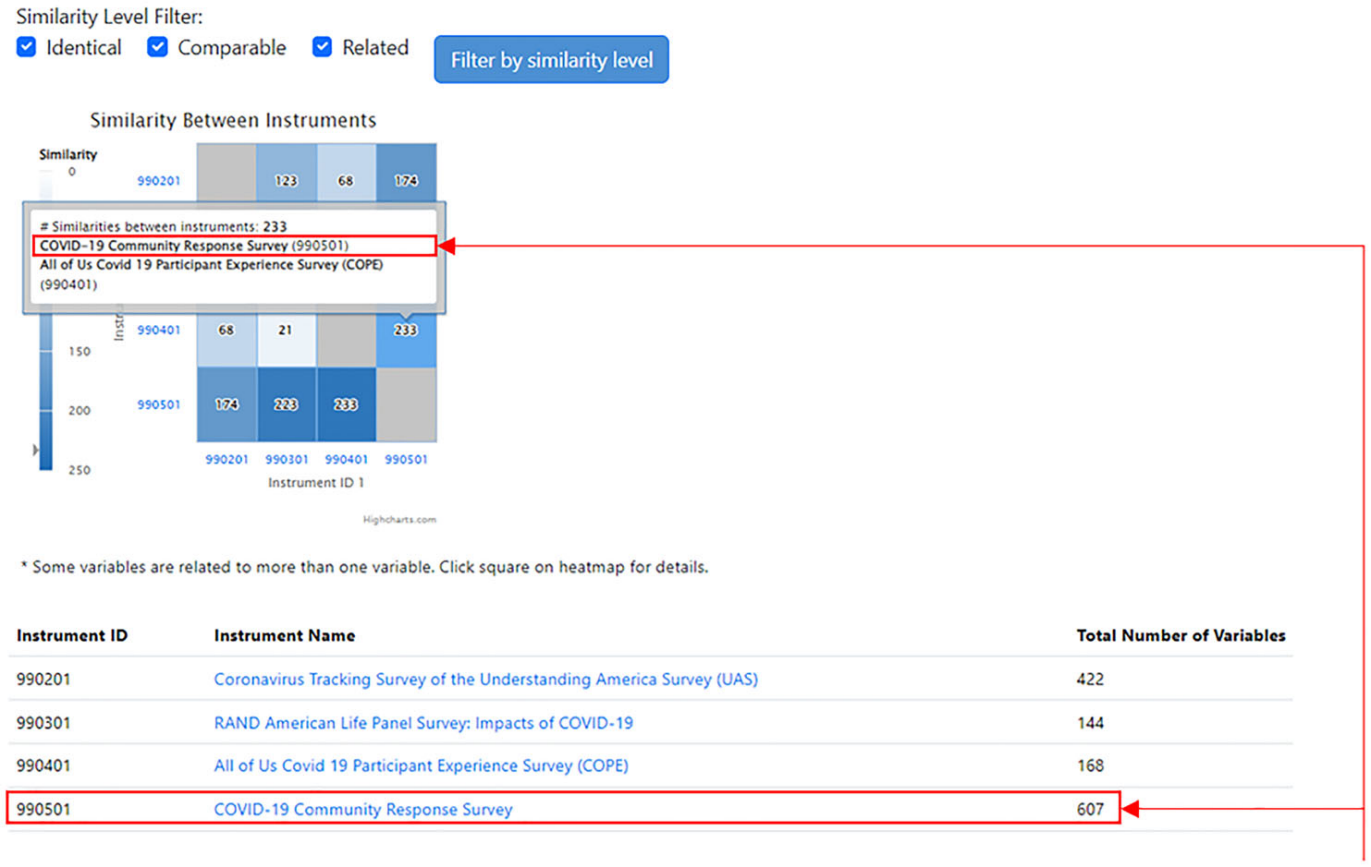
Heatmap and filtering results. A callout box will appear as the cursor hovers over any square in the heatmap. The callout box includes the number of similarities, and the names and IDs of the instruments compared.

Upon clicking to view all instruments or selected instruments, the results page will contain the following information: (1) additional filters for the similarity level (identical, comparable, or related) to refine the data displayed in the heatmap; (2) the heatmap itself; and (3) the instrument or protocol information on corresponding names, PDF links or links to the protocol in the PhenX Toolkit, and the number of variables, represented in a table below the heatmap. The X‐ and Y‐axis instrument ID labels link to the instrument PDF file or protocol. Hovering over a square on the heatmap shows the names of the instruments or protocols being compared (Fig. 10).

**Figure 11:**
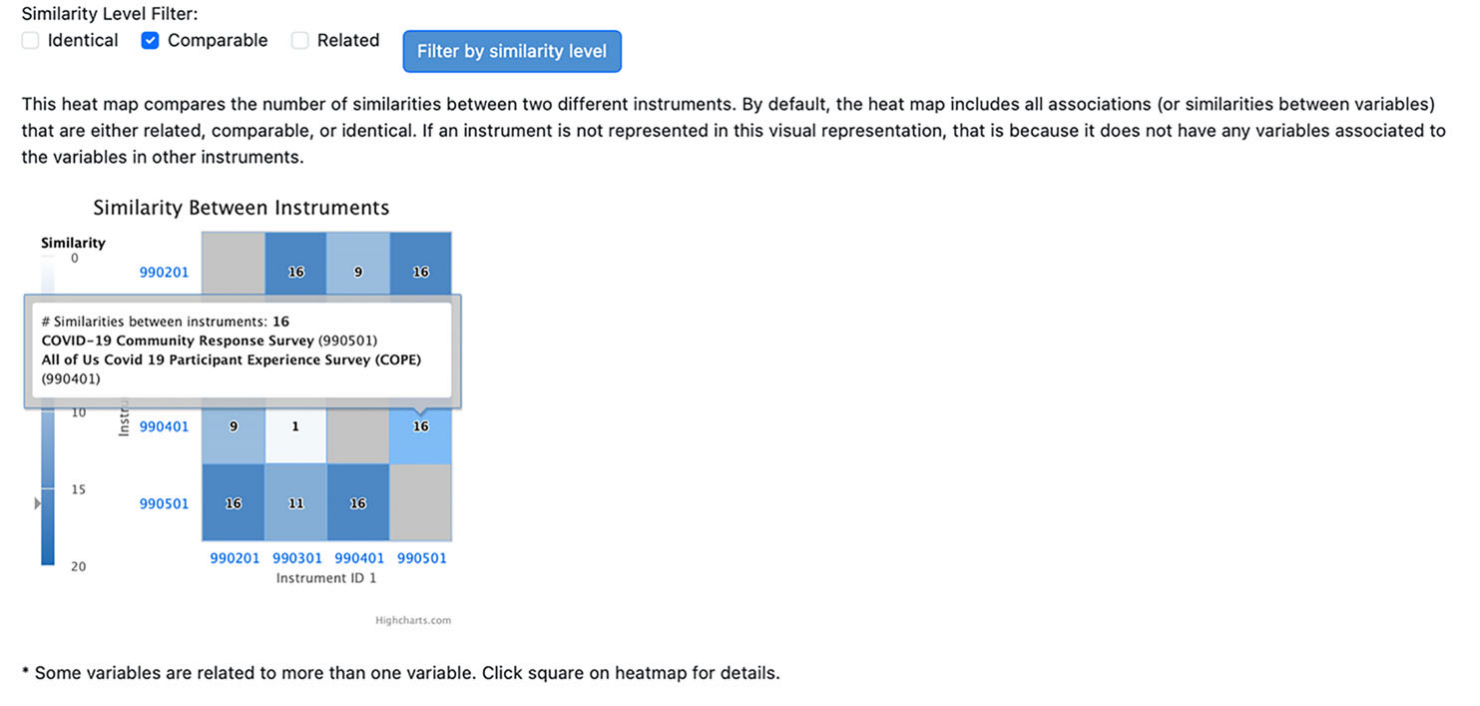
When a Similarity Level Filter (Comparable) is selected, the heatmap is updated to show only mappings at that similarity level.

A comparison of four protocols is shown in Figures 10 and 11: Coronavirus Tracking Survey of the Understanding America Survey (UAS), the RAND American Life Panel Survey: Impacts of COVID‐19, the “All of Us” Covid 19 Participant Experience Survey (COPE), and the COVID‐19 Community Response Survey. The assigned protocol ID for each is listed on the X‐ and Y‐axes. The square indicates there are 233 mappings between the two instruments (990401: “All of Us” Covid 19 Participant Experience Survey (COPE); 990501: COVID‐19 Community Response Survey), which includes mappings at the identical, comparable, and related levels. As illustrated in Figure 9A, if the investigator hovers over the intersection of similarities, a pop‐up box displays the number of mappings (233) and the names and IDs of the two instruments (990401: “All of Us” Covid 19 Participant Experience Survey (COPE); 990501: COVID‐19 Community Response Survey).

To get more specific information (e.g., number of mappings at the comparable level only), the investigator can deselect “Identical” and “Related” and update the filter by clicking the “Filter by similarity level” button. Using this filter, there are only 16 mappings between 990401: “All of Us” Covid 19 Participant Experience Survey (COPE) and 990501: COVID‐19 Community Response Survey (Fig. 11).

*NOTE*: Appropriate informed consent is necessary for obtaining and use of human study material.

## EXPLORING LONG COVID THROUGH COLLECTIONS AND THE VARIABLE COMPARE TOOL

The COVID‐19 Research Collection, the VCT, and the COVID‐19 Protocol Library may be used to influence study design, not only for COVID‐19 research, but also for all biomedical research studies to increase the FAIRification of collected data. FAIRification is the process of transforming, structuring, and preparing data that is consistent with the FAIR principles: Findable, Accessible, Interoperable, and Reusable (Tai et al., 2025). We illustrate the utility of the Long COVID Specialty Collection in combination with the COVID‐19 VCT by using the following scenario: A researcher is interested in conducting a study to review Long COVID symptoms and how they relate to quality of life. This could include symptoms during and after COVID‐19 and current living situation.

### Materials

Computer or laptop with internet access
Internet browser

**Figure 12:**
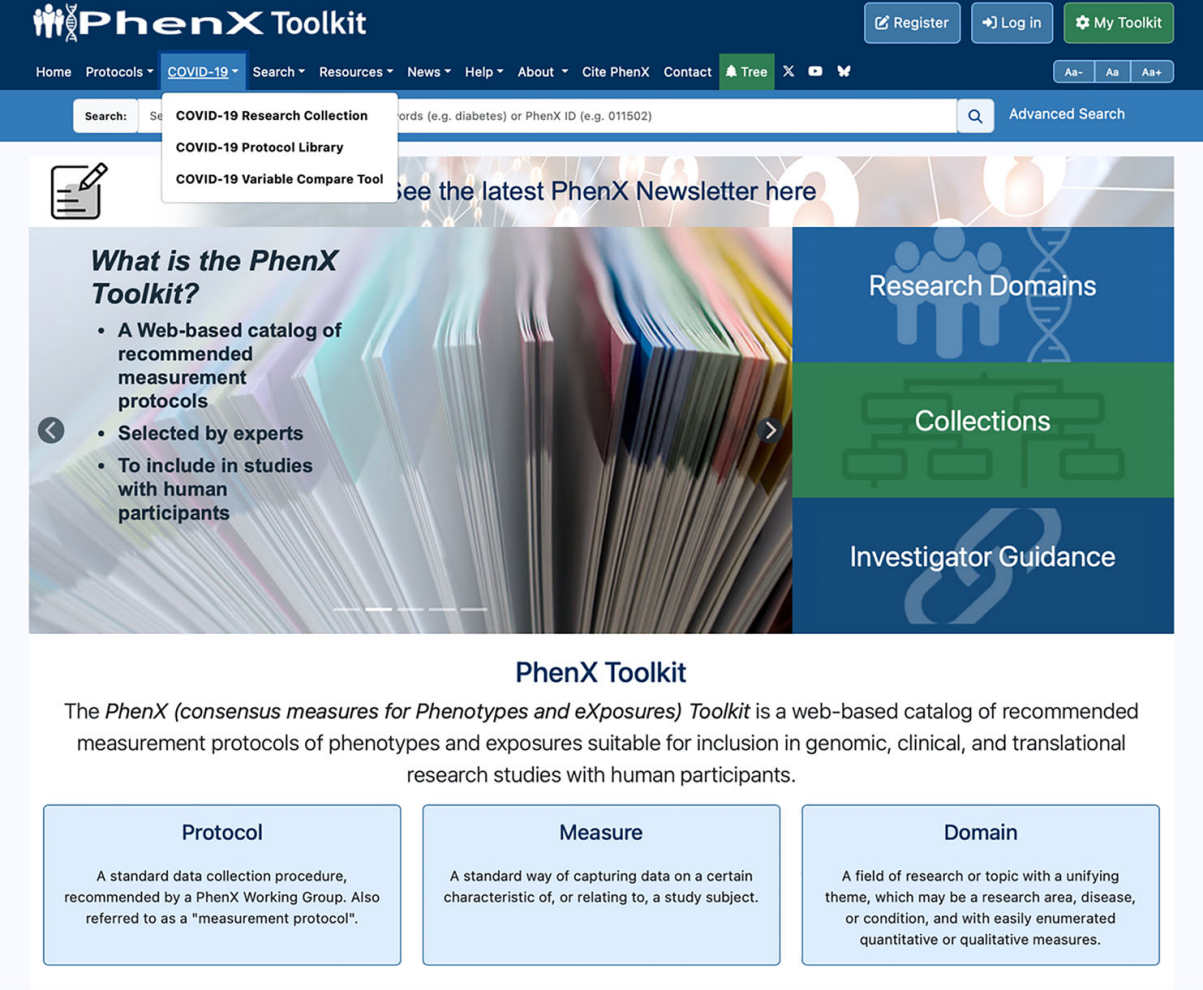
The PhenX Toolkit home page.

1 On your computer's internet browser, navigate to https://www.phenxtoolkit.org. View the research collections by clicking on “COVID‐19 Research Collection” from the “COVID‐19” dropdown in the top navigation menu (Fig. 12).

**Figure 13:**
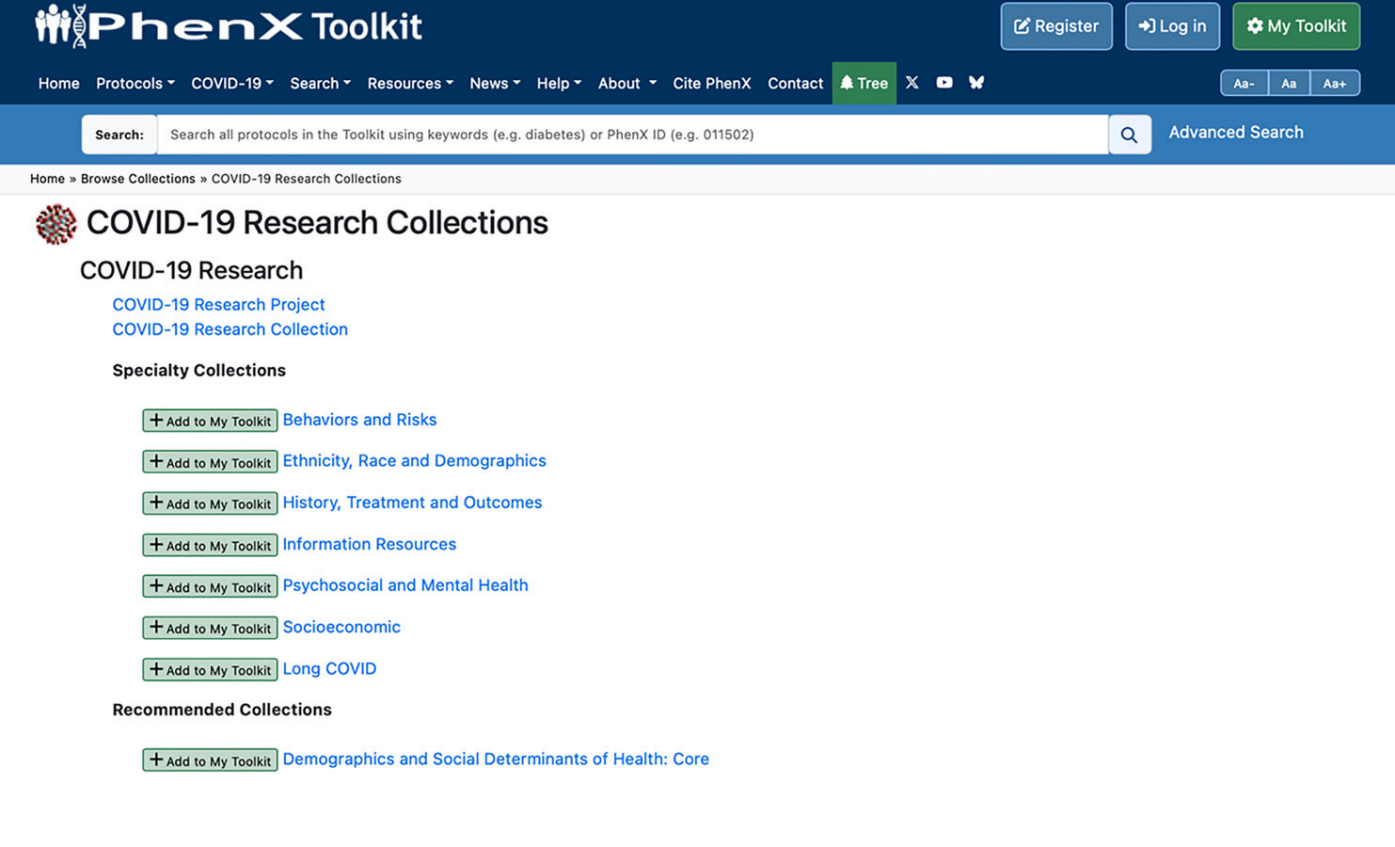
The PhenX Toolkit COVID‐19 Research Collection landing page.

2 Navigate to the Long COVID Specialty Collection (Fig. 13).

**Figure 14:**
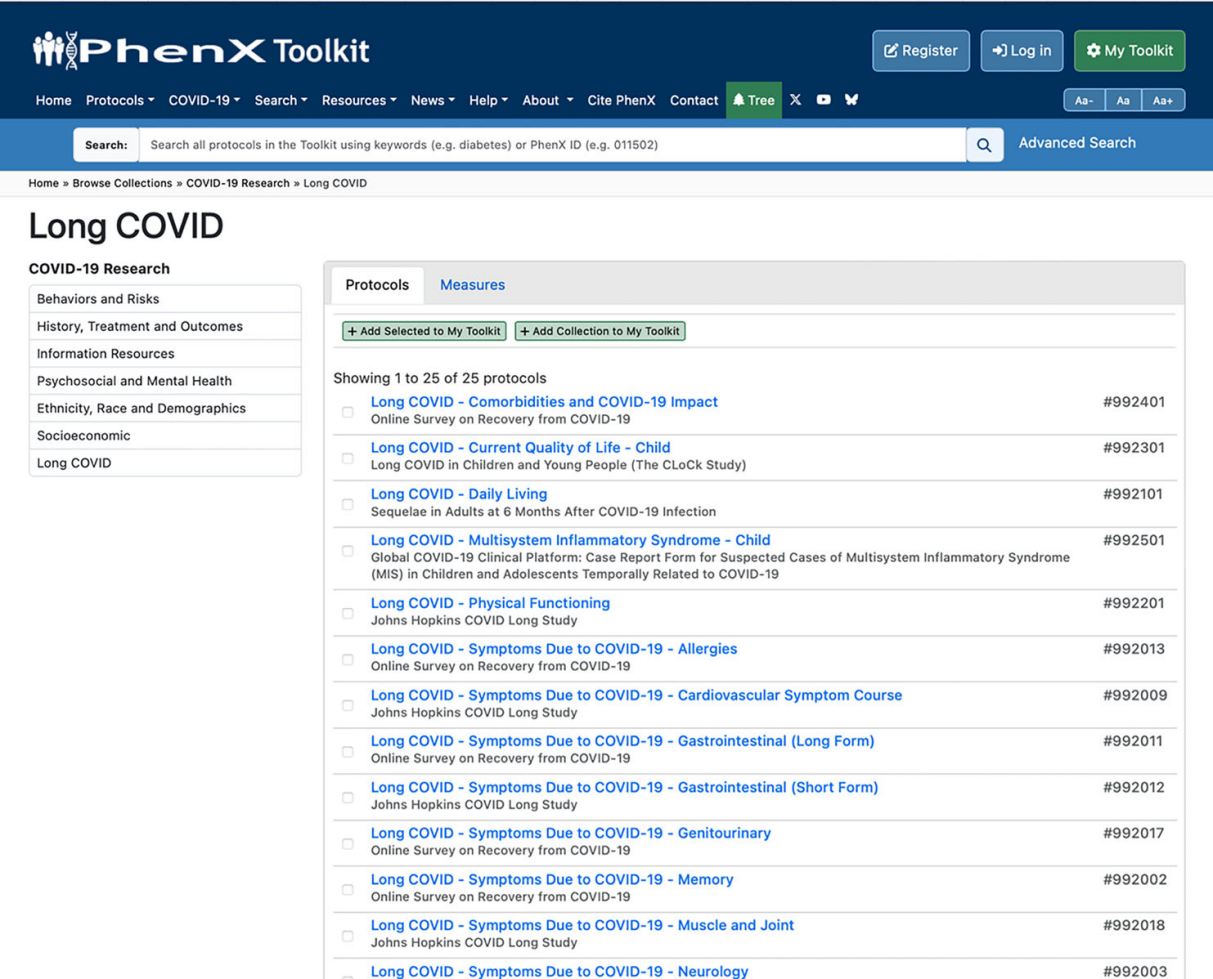
Long COVID Specialty Collection.

3 Browse Long COVID protocols (Fig. 14).
  Depending on their research needs, investigators can customize their toolkit by returning to the COVID‐19 Research Collection page (Fig. 13) and adding more collections that might be of interest.

**Figure 15:**
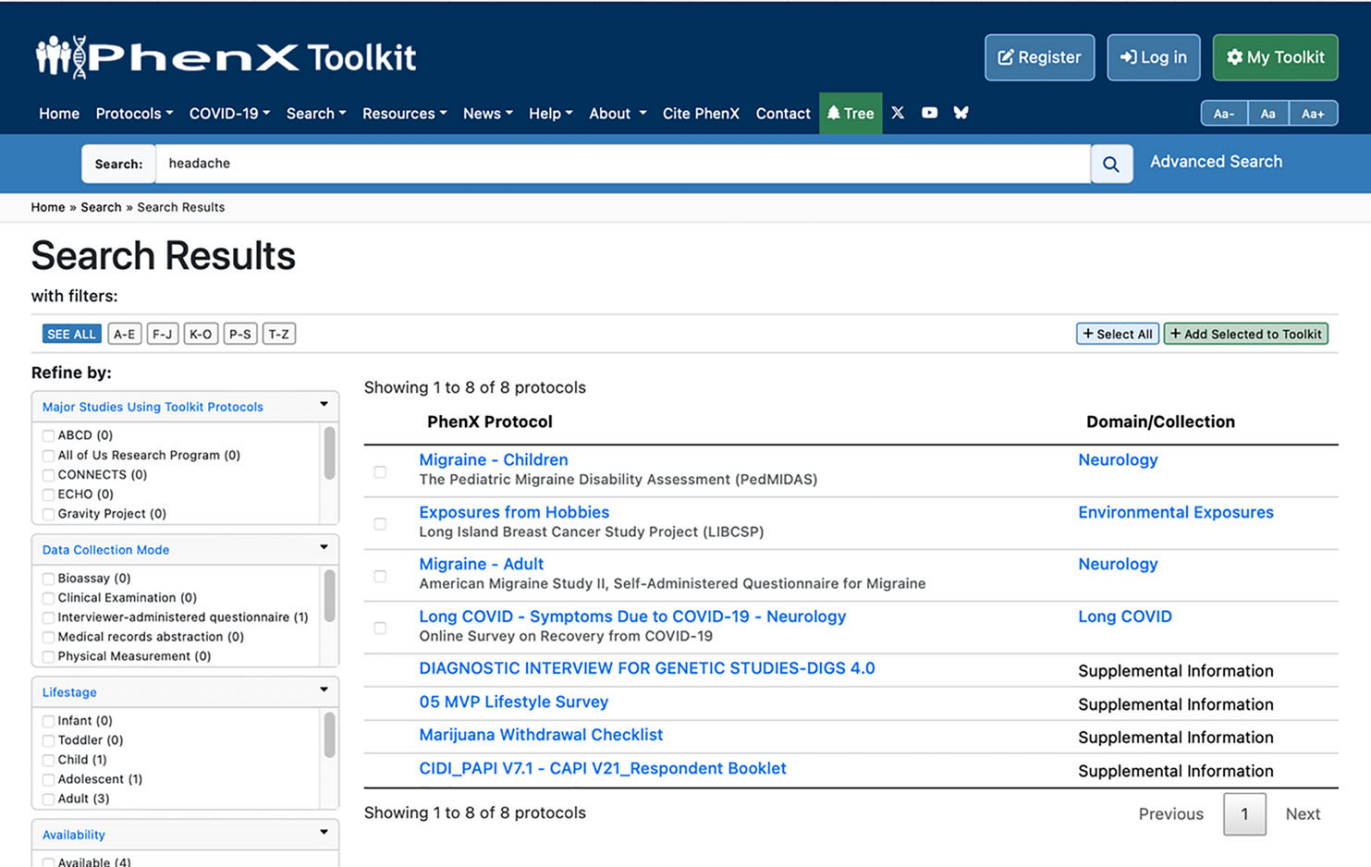
Search results using the keyword “headache.”.

4 Search the entire Toolkit for additional measurement protocols (Fig. 15).
  Optional: If investigators do not identify a collection of interest by name alone, they can use the search bar at the top of every page to look for specific measurement protocols.

**Figure 16:**
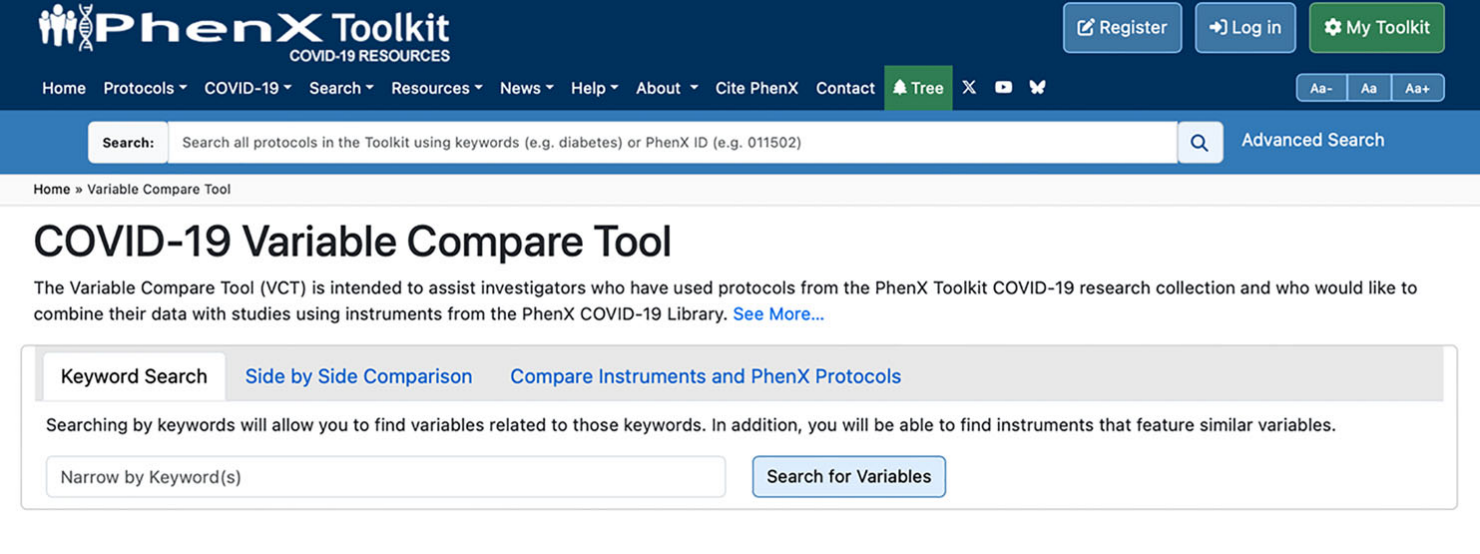
PhenX COVID‐19 Variable Compare Tool.

5 To search for additional COVID‐19 instruments that are already in use, use the VCT (Fig. 16). If the investigator is curious about other instruments that may cover headache symptoms, which is part of the “Long COVID ‐ Symptoms Due to COVID‐19 ‐ Neurology” protocol.

**Figure 17:**
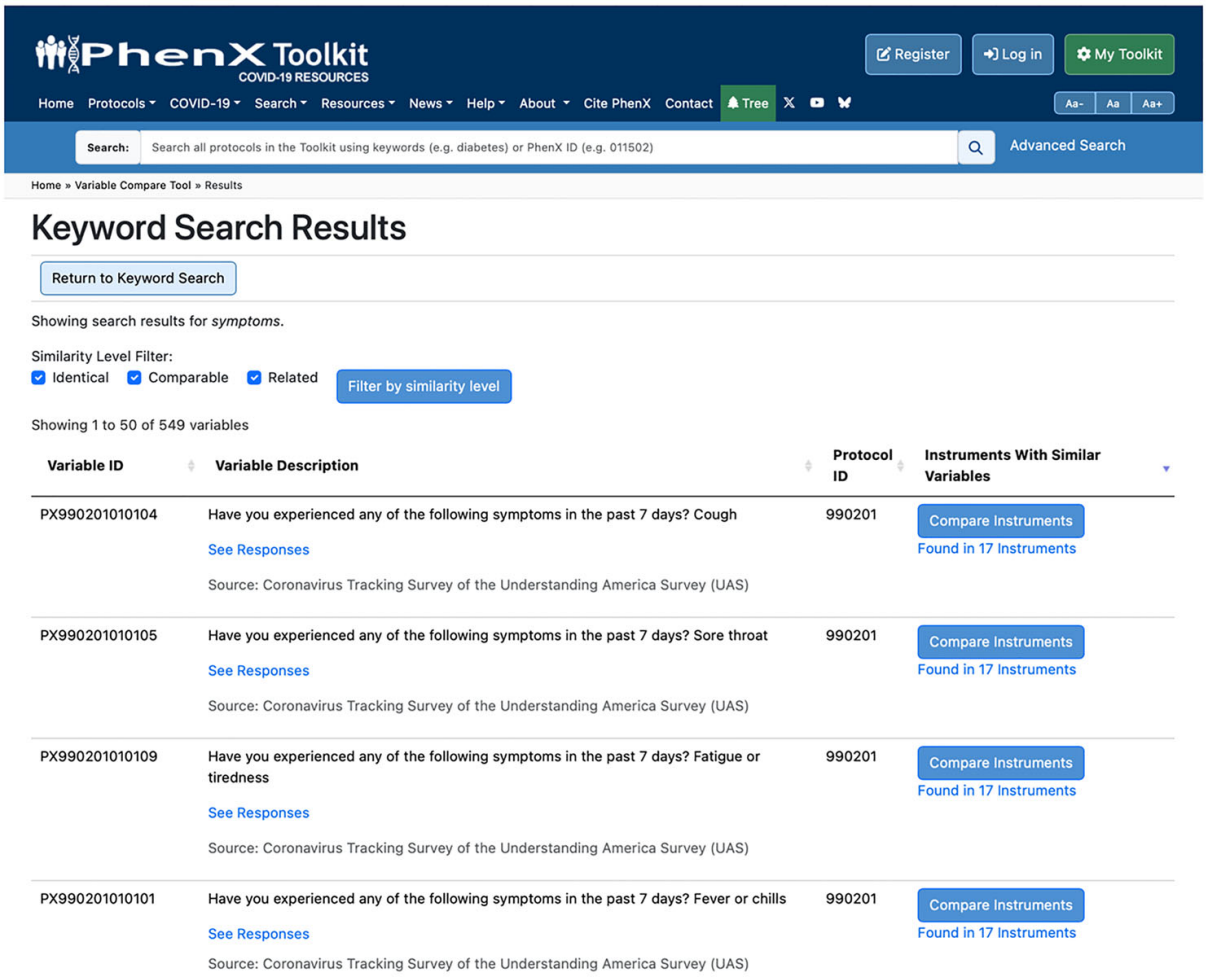
Example result from a keyword search with the term “symptoms.”.

6 Use the Keyword Search function, then type “symptoms” into the Keyword Search box to gain more insight into additional instruments containing questions related to symptoms. The results return 549 variables (Fig. 17).

**Figure 18:**
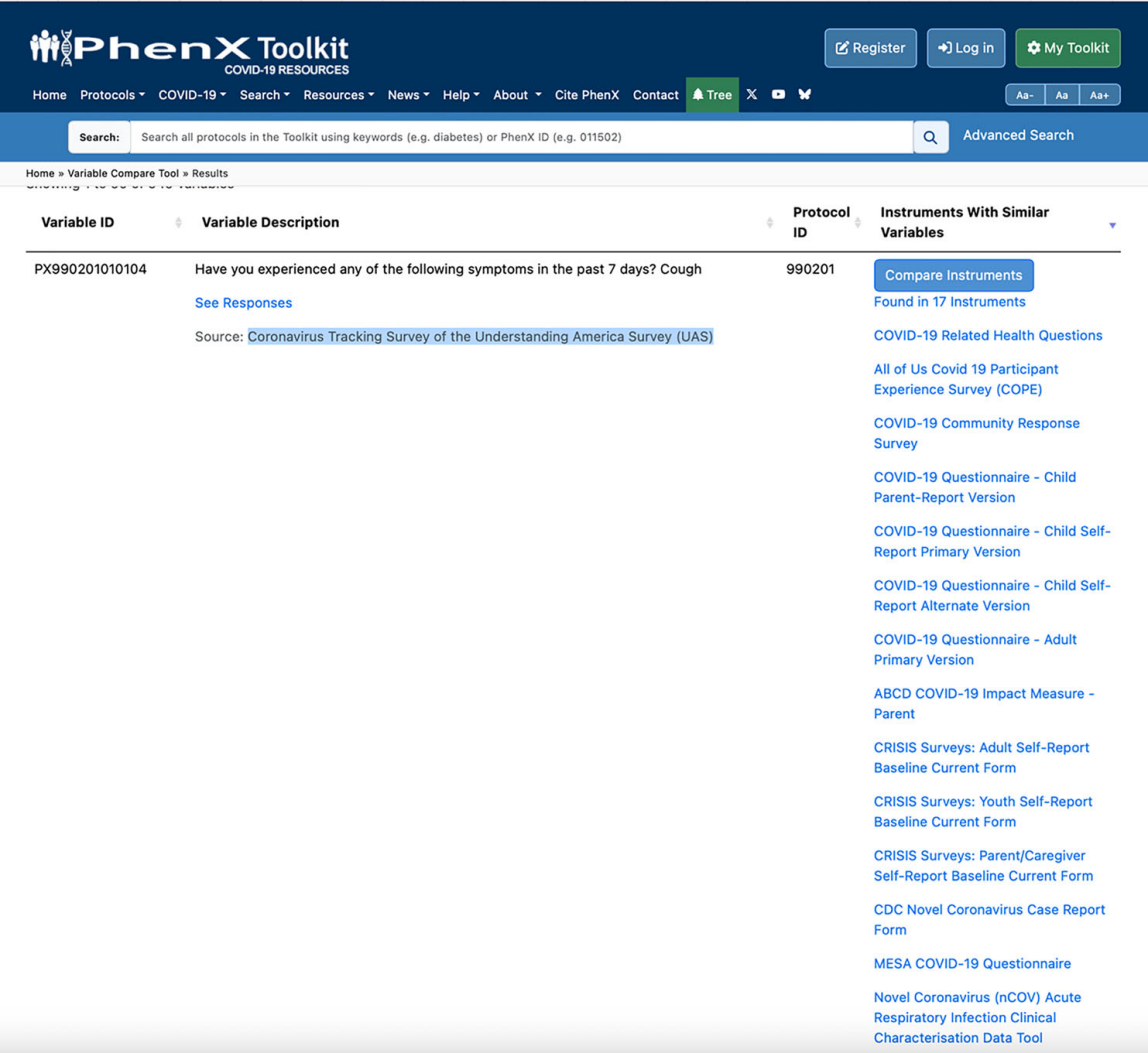
Expanded results from Figure 16.

7 Click “Found in 17 Instruments” to see which full instruments have variables similar to this variable from the Coronavirus Tracking Survey of the Understanding America Survey (UAS) (Fig. 18).

**Figure 19:**
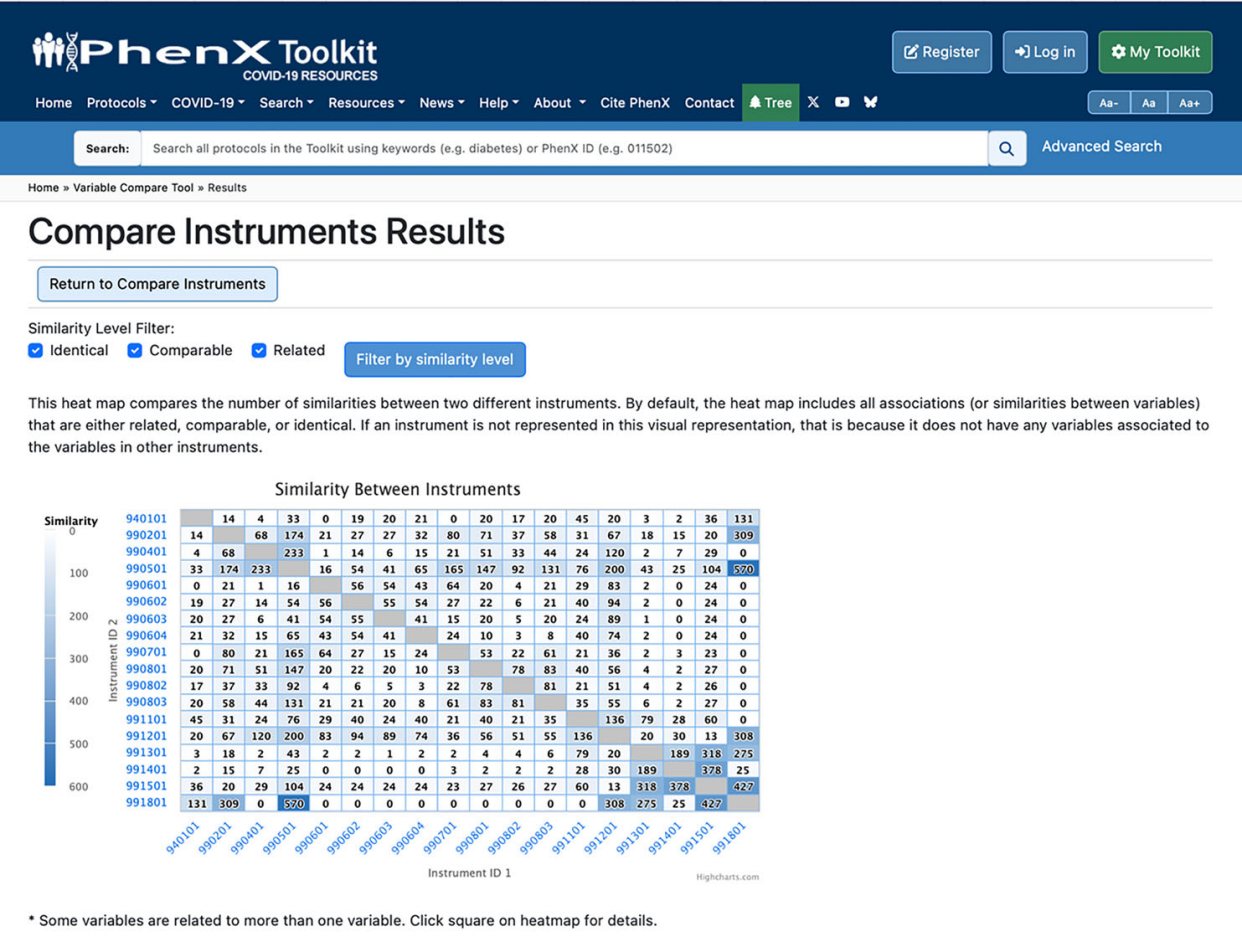
Results comparing the 17 related instruments with Coronavirus Tracking Survey of the Understanding America Survey (UAS).

8 Click Compare Instruments to see the degree of overlap across the 18 instruments listed (Fig. 19).

**Figure 20:**
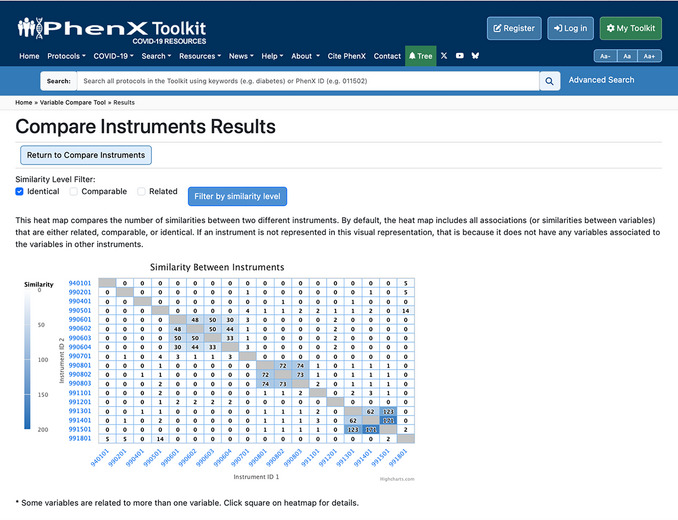
Results when the Identical filter is selected.

9 Check the “Identical” filter above the heatmap to see only the number of identical variables between full instruments (Fig. 20).

**Figure 21:**
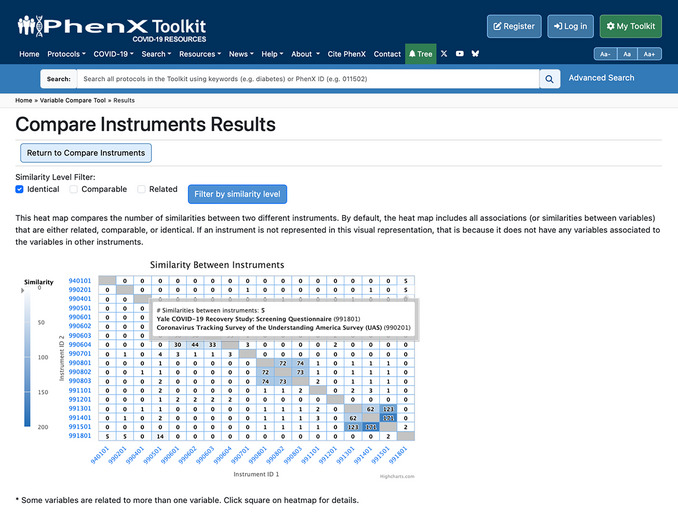
Hover box that appears showing the number of similarities and the names of the two instruments being compared.

10 Hover over the square showing the number of identical matches between instrument IDs 990201 and 991801. The square shows five identical matches between Coronavirus Tracking Survey of the Understanding America Survey (UAS) and Yale COVID‐19 Recovery Study: Screening Questionnaire (Fig. 21).

**Figure 22:**
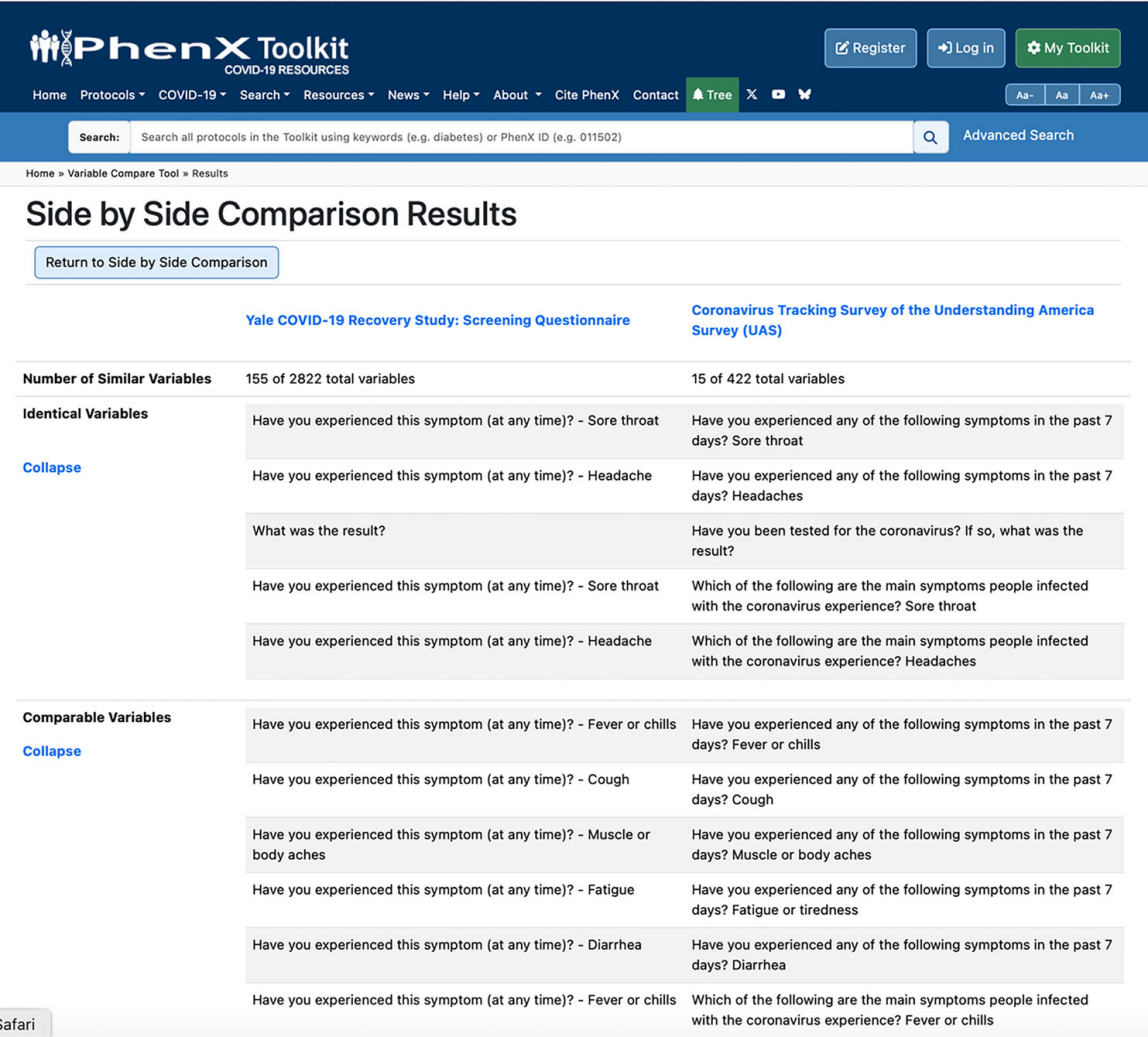
Side‐by‐Side Comparison results between Coronavirus Tracking Survey of the Understanding America Survey (UAS) and Yale COVID‐19 Recovery Study: Screening Questionnaire.

11 Click the square, which takes them to the Side‐by‐Side Comparison Results between Coronavirus Tracking Survey of the Understanding America Survey (UAS) and Yale COVID‐19 Recovery Study: Screening Questionnaire (Fig. 22).
  NOTE: One variable from the Yale COVID‐19 Recovery Study: Screening Questionnaire and two variables from the Coronavirus Tracking Survey of the Understanding America Survey (UAS) are related to symptoms of headache. This indicates at least two cases of similarity between these two questionnaires and suggests the two could be used to conduct an analysis cross‐study, in addition to the “Long COVID ‐ Symptoms Due to COVID‐19 ‐ Neurology” protocol from the Long COVID Specialty Collection, which also asks questions related to headache symptoms.
  A given variable from one full instrument can be mapped to multiple variables in another full instrument, so the numbers for each full instrument will not always be identical.

**Figure 23:**
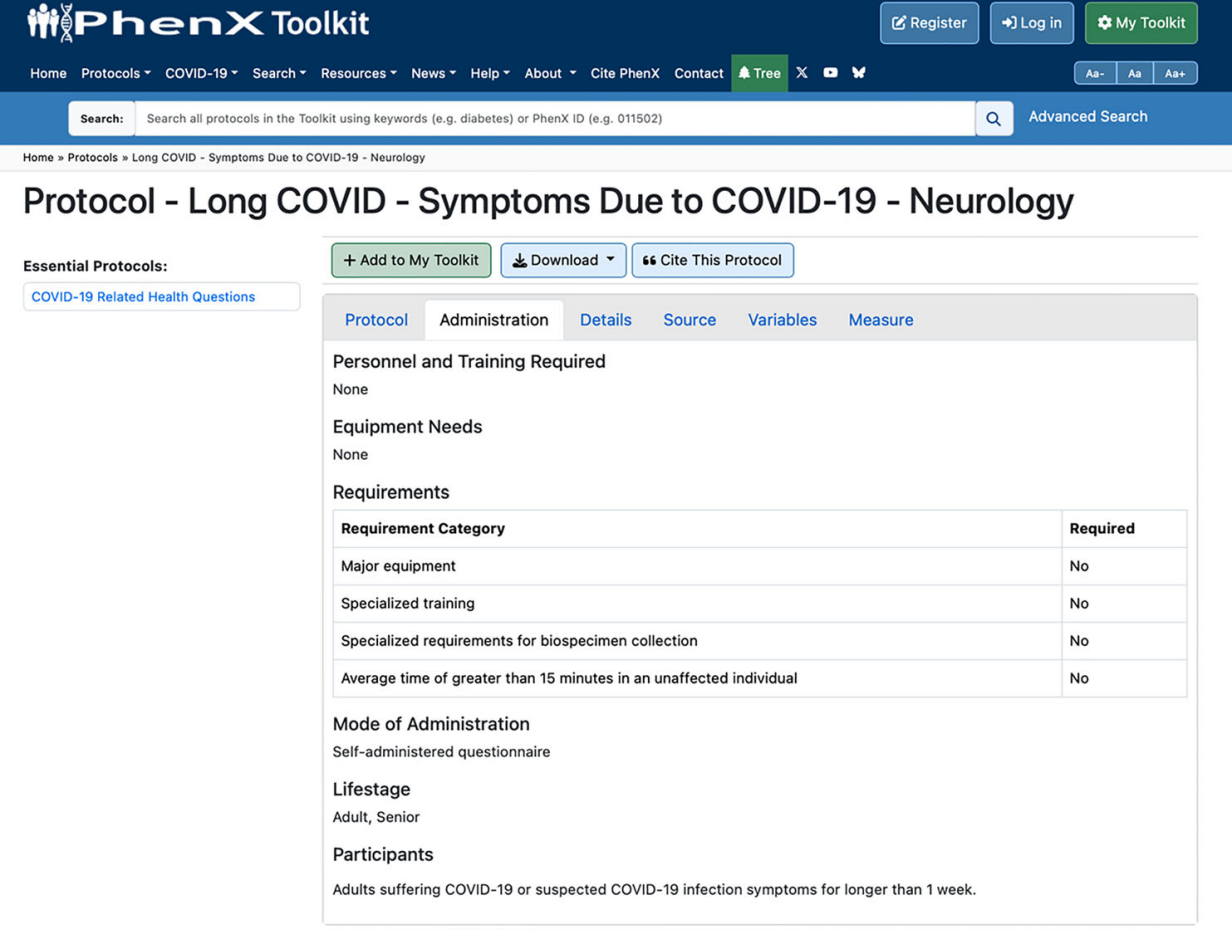
Requirements and participant details for the administration of PhenX protocols.

12 To obtain information needed to implement the protocol, click on the Administration tab on the Protocol page and review details which include training, equipment, suitability for specific life stages and other requirements (Fig. 23).

## COMMENTARY

### Critical Parameters

Basic Protocol explains how an investigator can browse and add protocols from the Long COVID Specialty Collection and how they can identify additional data collection instruments with cross‐analysis possibilities and explore the subject matter areas in other instruments using all PhenX COVID‐19 project resources. Although the PhenX Toolkit has the COVID‐19 Research Collection, we recognize these recommended protocols do not cover every aspect of the COVID‐19 pandemic, or investigators may be looking for something specific. Therefore, we encourage use of the COVID‐19 Protocol Library and the VCT to further expand the catalog of options.

The PhenX Toolkit is intended to support a variety of study designs. Because not all protocols are suitable for all study designs, decisions on which protocols to select are the investigator's responsibility. For the protocols included in the COVID‐19 Research Collection and the entire PhenX Toolkit, the Toolkit provides the following:

Related Protocols: Other protocols in the Toolkit that may be of interest (related protocols are suggestions only)
Essential Protocols: Required to correctly interpret selected protocols
DCW: Improves consistency of data collection
Data Dictionary (DD): Enhances data sharing and compatibility with REDCap.

For example, COVID‐19 Related Health Questions is considered essential to interpreting “Long COVID ‐ Symptoms Due to COVID‐19 – Neurology”. When an investigator selects “Long COVID ‐ Symptoms Due to COVID‐19 – Neurology”, they are encouraged to also select COVID‐19 Related Health Questions. The Toolkit also offers a DCW and a REDCap DD. The DCW helps investigators integrate PhenX protocols into their existing research and helps ensure that investigators collect the data needed for their measures. The DD describes the variables associated with the selected measures.

Due to the large number of variables and the small percentage that are similar to each other (4011/7869 = 51%) in the VCT, using Keyword Search and Side‐by‐Side Comparison may result in low‐yield results. Therefore, Basic Protocol may not be the best route for an investigator. We encourage investigators to explore the data underlying the VCT through multiple methods, such as viewing the Compare All Instruments heatmap first.

### Understanding Results

Since its launch in 2020, the COVID‐19 Protocol Library has had >51,000 views of instruments and module PDF files and >25,000 visits. 38 published articles have used COVID‐19 Protocol Library instruments. PhenX aims to assist those in the research community by periodically collecting feedback, running an informative webinar series, and providing researchers with guidance on using the PhenX Toolkit. Use of the COVID‐19 Research Collection of protocols and the COVID‐19 VCT will be more valuable over time, as investigators seek to combine data from different studies to increase statistical power or compare data from different studies to validate results.

For the PhenX COVID‐19 Research Collection, the PhenX team used a variation of the established PhenX consensus process to enable more rapid identification of high‐quality measures suitable for release in the Toolkit. The Long COVID Specialty Collection adds more recommended protocols, so investigators can combine and compare studies with increasing ease. Studies need to be compared to replicate results. In addition, combining studies increases the sample size, thus providing needed statistical power. Widespread adoption of PhenX measures will have a dramatic impact on biomedical research and ultimately on the health and well‐being of the population.

As illustrated in Figure 4, the number of similarities at the identical level (1016/7869 = 12.9%) and at the comparable level (1301/7869 = 16.5%) is low. When selecting candidate COVID‐19 library instruments for the VCT, we chose instruments from well‐known sources, such as WHO or the U.S. Census Bureau, or large studies, such as ECHO. We also included protocols from the COVID‐19 Research Collection in the VCT. Although there was some overlap, as indicated by the non‐zero percentage of identical or comparable variables, several instruments and all protocols were topic specific, decreasing the chance of mapping between variables. For example, data collection instruments covered different topic areas related to the COVID‐19 pandemic; some focused on symptoms and treatments related to COVID‐19, whereas others focused on socioeconomic impact due to social distancing measures. However, similar topics were still covered across this selection of instruments and protocols: 42.5% (3344/7869) of variables were linked at the related level, indicating that similar topic areas were heavily focused on COVID‐19 instruments. A larger pool of instruments in the VCT would increase the probability of identical and comparable variables and therein the usability and value of the tools, providing methods for harmonizing across instruments.

Although the classification of variable mappings as identical, comparable, and related is helpful, many challenges are associated with cross‐study analysis. Even for identical and comparable variables, the investigator needs to be confident that the context of the protocol is sufficiently similar to support combining data at the variable level. For related variables, the situation is even more complicated. That is, there are multiple ways to approach the harmonization of data from protocols and variables that map as related. In a best‐case scenario, two conceptually related protocols are both included in a study, thus making it possible to compare the results and perhaps develop an algorithm to enable combination or comparison of the data. Other approaches include the creation of derived variables or the comparison of total scores, but these methods require going beyond the individual variable level.

The VCT could evolve to be a resource that makes COVID‐19‐related data harmonization methods available to the scientific community. PhenX routinely identifies and assesses publications citing PhenX. As COVID‐19 cross‐study analyses are published, the PhenX team could extract and share methods, thus providing further support of the VCT for the scientific community.

### Time Considerations

The time required to collect data on a particular measure is addressed in the Requirements Table (see Fig. 5) for each specific protocol. Time, as it pertains to use of the PhenX Toolkit, is difficult to estimate. It could take as little as 1 hr for an investigator to select a few measures and request a report, DCW, or DD. However, it is more likely that an investigator would spend 1 to 2 hr becoming familiar with the Toolkit, select a few measures, then return to the Toolkit later to select additional measures. The PhenX Toolkit should be used as an integral part of developing a new study.

### Author Contributions

**Michelle Krzyzanowski**: Conceptualization; investigation; methodology; software; supervision; writing—original draft. **Helen Pan**: Conceptualization; data curation; methodology. **Iris Glaze**: Software; visualization; writing—original draft. **Stephen Hwang**: Conceptualization; data curation; methodology. **David Williams**: Data curation; methodology; writing—review and editing. **Michelle Engle**: Validation; visualization; writing—original draft. **Justin Waterfield**: Data curation. **Cataia Ives**: Data curation. **Sara Armson**: Data curation. **Wayne Huggins**: Conceptualization; methodology; visualization. **Carol M. Hamilton**: Conceptualization; investigation; methodology; writing—review and editing.

### Conflict of Interest

The authors declare no conflict of interest.

## Data Availability

The data that support the protocol are available in the PhenX Toolkit at https://www.phenxtoolkit.org/ or in the PhenX COVID‐19 Protocol Library https://www.phenxtoolkit.org/covid19.
